# Human Perivascular Adipose Tissue as a Regulator of the Vascular Microenvironment and Diseases of the Coronary Artery and Aorta

**DOI:** 10.29245/2578-3025/2019/4.1174

**Published:** 2019-08-13

**Authors:** Caitlin Stieber, Kimberly Malka, Joshua M. Boucher, Lucy Liaw

**Affiliations:** Center for Molecular Medicine, Maine Medical Center Research Institute, United States

**Keywords:** Perivascular adipose tissue, Cardiovascular disease, Secretome, Paracrine, Signaling

## Abstract

Perivascular adipose tissue (PVAT) is an adipose depot that surrounds blood vessels in the human body and exerts local paracrine signaling. Under physiologically healthy conditions, PVAT has an anti-contractile effect on vessels, but in obesity this effect is lost. During metabolic disease, adiponectin secretion is dysregulated, influencing nitric oxide bioavailability and macrophage infiltration and inflammation, all of which mediate PVAT signaling. However, based on the location in the body, and the type of adipocyte present, PVAT has different relationships with risk factors for disease. Imaging studies in patients with cardiovascular disease have demonstrated important associations between PVAT structure and pathology, yet insight into molecular pathways regulating human PVAT function are still lacking. This review focuses on our current understanding of human PVAT and its secretory role in the vascular microenvironment. A current area of priority is defining molecular differences in the secretome between PVAT depots, as this could inform the treatment of diseases that occur in anatomically restricted locations. In addition, understanding progressive changes in PVAT structure and function during metabolic disease is required for effective targeted therapies.

## Introduction

Blood vessel health is impacted by circulating and mechanical factors, which are integrated locally via distinct cellular interactions within the vascular microenvironment. One important component of this microenvironment, perivascular adipose tissue (PVAT), surrounds most major blood vessels in the human body and is comprised of adipocytes, macrophages, T-cells, collagen fibers, nerves, and capillaries^1^. PVAT is a secretory organ that exerts regulatory effects on the vasculature, depending on its location, type of adipocyte present, and if present in a healthy or diseased state. In mice, PVAT surrounding the thoracic aorta is similar to metabolically active brown adipose tissue (non-shivering temperature regulation via heat generation) whereas PVAT surrounding the abdominal aorta is similar to white adipose tissue (lipid storage function)^1,2^. Given the differences in adipocyte phenotype in distinct anatomical regions of PVAT in mice, it is presumed that similar variability may be present in humans. PVAT supports a vasorelaxation response in metabolically-healthy persons, promoting normal blood flow, and controlling blood pressure, while it has a pro-contractile activity in obese individuals^3–5^. This dysfunctional change in PVAT activity is thought to be due to changes in nitric oxide (NO) availability, oxidative stress due to macrophage infiltration, changes in vasoactive factors (such as hydrogen peroxide or hydrogen sulfide) and release of other unknown factors^6^. Excess vasocontraction could lead to hypertension and increased risk for vascular disease. The majority of the information regarding signaling between PVAT and the underlying vessel wall has come from studies in rodent models. Parallel studies in humans are challenging and lacking due to the highly invasive nature of PVAT procurement (e.g. located on ascending aorta, carotid arteries) ^6,7^. The purpose of this mini-review is to summarize our knowledge of human aortic and pericoronary PVAT in healthy and disease states with a focus on paracrine secretion, and highlight imaging techniques that have allowed for less invasive analysis of human PVAT and correlations to cardiovascular diseases (CVD) that are impacted in these regions, including coronary atherosclerosis and aortic aneurysms.

## PVAT Contributions to Human CVD

PVAT function and its effect on CVD differs based on the location of the vessel it surrounds, thus this discussion will be separated by anatomical location. The Framingham Heart Study has provided unique insight into associations between PVAT, risk factors for CVD, and vascular function. In metabolically healthy individuals, the volume of thoracic peri-aortic PVAT is positively correlated with body mass index (BMI), waist circumference, hypertension, lower HDL, serum triglycerides, impaired fasting glucose and diabetes, adiposity, metabolic risk factors, coronary and abdominal aortic calcification^7^. In addition, a positive correlation was also found between PVAT deposition around the thoracic aorta and peripheral artery disease^8^. Further analysis to evaluate thoracic peri-aortic PVAT as a risk marker for CVD showed that increased volume of thoracic PVAT is frequent in a community sample, and high PVAT without high visceral adipose tissue was found in individuals with a greater prevalence of CVD^9^. Further study determined that higher than average volumes of PVAT around the thoracic aorta was negatively associated with microvascular function and corresponded with increased arterial stiffness^10^.

The relationship is less clear in coronary PVAT, and several studies found that increased volumes of coronary PVAT are positively correlated to the presence and size of plaques in coronary atherosclerosis^7,11,12^. In opposition, Gorter et al. found no relationship between PVAT surrounding coronary arteries and severity of coronary atherosclerosis^13^. However, in patients with low BMI but multivessel coronary artery disease, coronary PVAT thickness was increased^13^. Additionally, Lesna et al. found no difference in coronary PVAT area between coronary heart disease (plaques present) and dilated cardiomyopathy (no plaques), suggesting no association between PVAT and coronary atherosclerosis^14^. Discrepancies in findings may be due to the design of studies with limited sample sizes or cross-sectional designs limiting conclusions. Finally, the expected variability in human samples and analyses that do not distinguish between different cardiovascular pathologies add complexity in interpretation.

In terms of the abdominal compartment, multiple studies in mice have shown that PVAT contributes to abdominal aortic aneurysm, an abnormal breakdown of the vascular wall that increases the risk of rupture, by recruiting immune cells into the adventitia of the aorta^15,16^. Immune cell recruitment to this region was associated with increased IL-6, cathepsins, and MMPs, suggesting a proteolytic environment. Additionally, limited studies in humans have shown that PVAT surrounding aneurysmal tissue has higher levels of inflammatory markers than PVAT in patients with no aneurysmal disease^16^. Molecular profiling studies demonstrate a difference in gene expression in PVAT surrounding aneurysmal aortic tissue when compared to PVAT surrounding the healthy, non-dilated aorta in the same patient^17^. Studies in human tissue have found links between diseased PVAT and vascular smooth muscle cell phenotype; however, there are no studies that demonstrate a direct effect of PVAT on vascular smooth muscle cells in human tissues^18,19^. Ongoing and future research with patients requires subclassification by specific vascular pathology, along with continued molecular characterization of anatomically distinct PVAT depots during disease progression.

## Imaging Methods to Evaluate Human PVAT

Recent correlation of PVAT volume and phenotype with CVD has highlighted imaging techniques as potential diagnostic or surveillance tools. Extra media-thickness is an ultrasound index that is a non-invasive measure of PVAT which allows examination of the relationship between the adipose tissue and cardiometabolic risk^20^. Computed tomography (CT) allows detection of PVAT surrounding coronary arteries and plaque burden within the arteries at the same time, which allows examination of how the relationship between PVAT and blood vessels change during atherosclerosis^21,22^. CT has also been used to demonstrate a higher density of PVAT around abdominal aortic aneurysms compared to the adjacent normal vessel segment^23^. CT data are also used to derive the fat attenuation index (FAI), which is a biomarker of coronary inflammation. FAI is positively correlated with atherosclerotic plaque burden, and is inversely correlated with adipogenic gene expression^22,24^. High FAI on the right coronary artery and left anterior descending artery predicted all cause and cardiac mortality, leading to the conclusion that FAI is a better predictor of mortality than previous biomarkers including extra media-thickness^24^. Magnetic resonance imaging has been used to distinguish between the fat and the aortic wall, showing a weak correlation between PVAT thickness and aortic wall thickness. These data published by Alkhalil et al. do not support the conclusions of other studies of a positive correlation of PVAT volume and disease^25^. These contrasting data show variability within the limited body of work on human PVAT, and highlight the need for additional studies.

## Secretory Effects of PVAT

PVAT exerts paracrine effects on the surrounding vasculature^7^, and adipocytes influence tissue through the secretion of adipokines^26^. Adiponectin is the predominant mediator of vasorelaxation in humans, and thus its relationship with PVAT is being investigated^4^. Adiponectin has anti-inflammatory and insulin sensitizing functions^27^ and has been implicated as a risk factor in CVD. Dysfunctional adipocytes secrete less adiponectin, and promotion of healthy adipogenesis may rescue this deficit^6^. Despite its protective effect, patients with a BMI above 30 had increased adiponectin secretion from PVAT^6^, as did symptomatic patients with carotid stenosis^28^ and patients with wound complications defined as any type of surgical site infection, dehiscence, or other (seroma, lymphocele, hematoma)^29^.

In patients who have undergone bariatric surgery, the levels of adiponectin secreted from PVAT are higher 6 months after surgery, along with restoration of the anti-contractile activity of PVAT, indicating that adiponectin restoration is associated with vasoprotection^3^. Secretion of adiponectin has also been studied using *ex vivo* analysis of PVAT from stenotic or non-stenotic coronary artery segments from patients undergoing coronary artery bypass grafting. There were no differences in adiponectin secretion from these PVAT samples following 24h conditioning of medium, which could be a difference either in anatomical derivation of the PVAT or due to the *ex vivo* nature of the study^12^.

Adiponectin increases nitric oxide synthase activity^4^ and there is a positive association between PVAT adiponectin, NADPH oxidase-generated reactive oxygen species, and endothelial nitric oxide synthase uncoupling^27,30^. Although the PVAT and endothelium functionally overlap in their production of vasoactive compounds such as NO and prostaglandins, studies in the absence of endothelium or PVAT have shown that each has pertinent function of vascular contractility (recently reviewed^31^). Increasing adiponectin and NO availability restores PVAT anti-contractile activity that is lost in obesity^3^. Hypoxia and inflammation reduce adiponectin production^4^. In obesity, adiponectin production is decreased, and attributed to NO inhibition^5^. Furthermore, reduced adiponectin in Type 2 diabetes stimulates NADPH oxidase, which is sensed by PVAT causing an upregulation in adiponectin^27^, which is a possible explanation of increased adiponectin in obesity and other disease states.

This body of evidence indicates a paradoxical activity of adiponectin, which is usually protective, but its expression seems to be increased in diseased PVAT^28^. In most of these disease/injury states, it would be expected that adiponectin would decrease given an increased number of dysfunctional adipocytes. Since the opposite is true, it has been hypothesized that more healthy adipocytes are produced in disease states as a rescue mechanism, which may implicate activation of adipocyte progenitor cells. In many of these cases, only the adiponectin levels secreted from PVAT were affected, rather than overall circulating levels, indicating regulation within the local microenvironment.

## Macrophages and Inflammation

Macrophages and T lymphocytes accumulate between the PVAT and adventitia of atherosclerotic aortas^32^. This is also true of PVAT surrounding sites of vascular injury due to abdominal aortic aneurysms^16^. Inflammatory infiltration in PVAT seems to favor M1 macrophages that are pro-inflammatory^33^. In PVAT surrounding stenotic arteries, there are more macrophages than in PVAT surrounding control arteries. However, there is no difference in macrophage infiltration and M2 macrophages are the most abundant in both cases, challenging the hypothesis that M1 macrophages are favored. There is increased expression of pro-inflammatory cytokines (IL-1α, IL-17, IL-18, IL-23) and an anti-inflammatory cytokine (IL-5) in PVAT surrounding stenotic arteries, and this pro-inflammatory effect is dampened in coronary artery disease^12^. Consistent with the pro-inflammatory hypothesis in disease states, cathepsin S, which induces macrophage migration, is elevated in PVAT surrounding aortic aneurysms^16^. In PVAT from patients with coronary artery disease, inflammatory genes were upregulated, suggesting a role for PVAT in atherosclerosis and indicating PVAT may have differing effects in different pathologies^34^. These observations of macrophage polarization in human PVAT during vascular disease are associative, and more study is required to understand implications to disease progression. It is interesting to consider that subsets of macrophages have also been defined in discrete locations within human atherosclerotic lesions^35^, and localization of inflammatory subtypes within vascular plaques may drive infiltration into adjacent PVAT.

## PVAT Progenitor Populations

To understand different phenotypes of mature PVAT, it is important to study PVAT progenitor cells. Work from our lab found cultured PVAT progenitors to lack CD45 and CD31 but express cell surface proteins CD73, CD90, CD105, and CD140A^36^. Based on the study of perivascular adipose stromal cells, PVAT progenitors can differentiate into myofibroblasts, endothelial cells, and adipocytes, but brown adipocytes were not detected^37^. Corroborating this, we found that human PVAT progenitors have adipogenic and chondrogenic potential, but lack osteogenic potential^38^. Additionally, perivascular preadipocytes secrete more inflammatory cytokines and chemokines compared to mature adipocytes^39^, highlighting the need to study both populations using cells derived from human PVAT.

Rab proteins regulate protein trafficking in secretion across all tissue/cell types in the body, and are important modulators of insulin sensitivity^40,41^ and adipose tissue function^42^. One family member, Rab27a, has recently been characterized in human PVAT-derived adipocytes^36^, and is involved in exosome secretion^43^. An area just beginning to be addressed is the pathway by which secretion is regulated in human PVAT. Using human PVAT-derived adipose progenitor cells, we recently showed that suppression of Rab27a decreased *in vitro* adipogenesis and lipid mobilization in monolayer cultured preadipocytes derived from human aortic PVAT. Because Rab27a regulates protein trafficking and vesicle secretion^43,44^, Rab27a may influence adipogenesis and inflammation via regulation of autocrine/paracrine factor secretion. There is evidence for this in a mouse model of Rab27a/Rab27b knockout, where there is deficient exosome secretion, leading to chronic, low-grade inflammation^45^. In humans, mutation in the *Rab27a* gene causes type 2 Griscelli syndrome, characterized by silver hair caused by defective melanosome transport, and immunodeficiency due to defective exocytosis in lymphocytes^46^. Recently, a case study described a young Grisselli syndrome patient with macrophage activation syndrome, which is characterized by expansion of macrophages and T cells, and an uncontrolled inflammatory response^47^. Given both secretory and non-secretory roles of Rab27a^36,48^ and its regulation of inflammation, more investigation is required to understand how Rab27a may control modulates adipose expansion and differentiation in human PVAT with relation to cardiovascular disease.

## Conclusion

**Figure 1.**
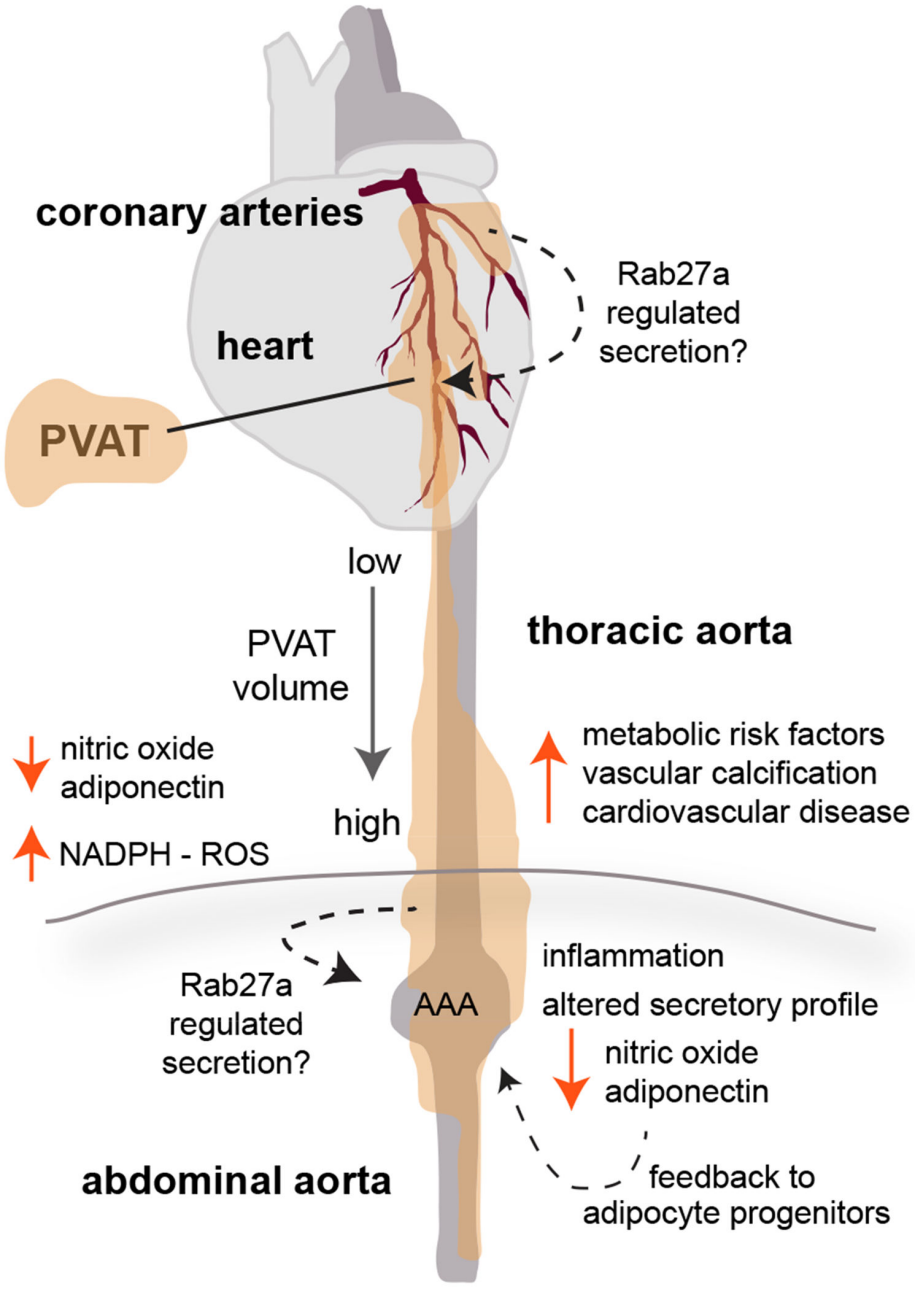
PVAT Influence On Human CVD. Characteristics of PVAT are distinct based on anatomical location. In humans, PVAT surrounding coronary arteries, thoracic aorta, and abdominal aorta have been studied. The volume of PVAT has been positively correlated to cardiovascular disease risk, with increasing PVAT volume surrounding the thoracic aorta associated with increased metabolic risk factors. Thus, clinical imaging techniques may be useful for diagnostics or monitoring. Signaling mechanisms by which human PVAT influences disease include modification of vasoregulatory pathways such as nitric oxide, adipokines, and reactive oxygen species (ROS). Although more studies are analyzing these pathways in patients and human tissues, most of our molecular signaling information has been derived from pre-clinical studies, particularly using mouse models. Those results have informed the following model of how alterations in PVAT in obesity may promote vascular disease: as PVAT volume increases and becomes more lipid-storing, there is decreased adiponectin and nitric oxide production, leading to increased vasoconstriction and generation of reactive oxygen species. Inflammation within PVAT is also associated with obesity and metabolic disease, and we predict that activation of inflammatory cytokines contributes to a feed-forward pathological loop. Our studies in PVAT suggest that the well-known trafficking and secretory molecule, Rab27a, may contribute to these processes by activation during obesity to change the content and/or rate of secretion of paracrine factors derived from adipocyte progenitors, mature adipocytes, or inflammatory cells within PVAT. This mechanism is currently under investigation. PVAT dysfunction has implications for coronary artery disease, arterial calcification, abdominal aortic aneurysm (AAA), and peripheral artery disease in humans.

There is increasing evidence that human PVAT plays a role in controlling the vascular microenvironment and susceptibility to vascular disease. However, unlike more advanced studies in rodent models, the literature with regards to human PVAT and CVD is not conclusive, although mechanisms are emerging (Figure 1). There is limited information about molecular characteristics of human PVAT cells, including adipocyte progenitors, mature adipocytes, and other stromal cells. In addition, although it is evident that PVAT has paracrine signaling activity to the vessel wall, mechanisms of regulated secretion are still under investigation. PVAT function differs based both on location and individual disease state, which highlights the need to examine multiple PVAT depots in several disease states in obese and non-obese patients. While much of this study has been limited due to the invasive nature of procedures to procure PVAT, *in vitro* assays with human PVAT-derived cells are feasible^36,38^, and continuing advances identifying PVAT markers and imaging methods are helping to resolve this issue. More detailed understanding of the secretory role of PVAT in regulating blood vessel function will be applicable to treatment of atherosclerosis, arterial calcification, aneurysms, and other CVD.
